# Sex Differences in Frequency, Severity, and Distribution of Cerebral Microbleeds

**DOI:** 10.1001/jamanetworkopen.2024.39571

**Published:** 2024-10-15

**Authors:** Simon Fandler-Höfler, Sebastian Eppinger, Gareth Ambler, Philip Nash, Markus Kneihsl, Keon-Joo Lee, Jae-Sung Lim, Masayuki Shiozawa, Masatoshi Koga, Linxin Li, Caroline Lovelock, Hugues Chabriat, Michael Hennerici, Yuen Kwun Wong, Henry Ka Fung Mak, Luis Prats-Sanchez, Alejandro Martínez-Domeño, Shigeru Inamura, Kazuhisa Yoshifuji, Ethem Murat Arsava, Solveig Horstmann, Jan Purrucker, Bonnie Yin Ka Lam, Adrian Wong, Young Dae Kim, Tae-Jin Song, Robin Lemmens, Ender Uysal, Zeynep Tanriverdi, Natan M. Bornstein, Einor Ben Assayag, Hen Hallevi, Jeremy Molad, Masashi Nishihara, Jun Tanaka, Shelagh B. Coutts, Alexandros Polymeris, Benjamin Wagner, David J. Seiffge, Philippe Lyrer, L. Jaap Kappelle, Rustam Al-Shahi Salman, Maria Valdes Hernandez, Hans R. Jäger, Gregory Y. H. Lip, Urs Fischer, Marwan El-Koussy, Jean-Louis Mas, Laurence Legrand, Christopher Karayiannis, Thanh Phan, Sarah Gunkel, Nicolas Christ, Jill Abrigo, Winnie Chu, Thomas Leung, Francesca Chappell, Stephen Makin, Derek Hayden, David J. Williams, Werner H. Mess, M. Eline Kooi, Carmen Barbato, Simone Browning, Anil M. Tuladhar, Noortje Maaijwee, Anne Cristine Guevarra, Anne-Marie Mendyk, Christine Delmaire, Sebastian Köhler, Robert van Oostenbrugge, Ying Zhou, Chao Xu, Saima Hilal, Caroline Robert, Christopher Chen, Min Lou, Julie Staals, Régis Bordet, Nagaendran Kandiah, Frank-Erik de Leeuw, Robert Simister, Daniel Bos, Peter J. Kelly, Joanna Wardlaw, Yannie Soo, Felix Fluri, Velandai Srikanth, David Calvet, Simon Jung, Vincent I. H. Kwa, Stefan T. Engelter, Nils Peters, Eric E. Smith, Hideo Hara, Yusuke Yakushiji, Dilek Necioglu Orken, Vincent Thijs, Ji Hoe Heo, Vincent Mok, Roland Veltkamp, Hakan Ay, Toshio Imaizumi, Kui Kai Lau, Eric Jouvent, Peter M. Rothwell, Kazunori Toyoda, Hee-Joon Bae, Joan Marti-Fabregas, Duncan Wilson, Jonathan Best, Franz Fazekas, Christian Enzinger, David J. Werring, Thomas Gattringer

**Affiliations:** 1 Department of Neurology, Medical University of Graz, Graz, Austria; 2 Stroke Research Centre, UCL Queen Square Institute of Neurology, London, United Kingdom; 3 Division of Neuroradiology, Vascular and Interventional Radiology, Department of Radiology, Medical University of Graz, Graz, Austria; 4 Department of Statistical Science, University College London, London, United Kingdom; 5 Department of Neurology, Korea University Guro Hospital, Seoul, South Korea; 6 Department of Neurology, Hallym Neurological Institute, Hallym University College of Medicine, Hallym University Sacred Heart Hospital, Anyang, South Korea; 7 Department of Cerebrovascular Medicine, National Cerebral and Cardiovascular Centre, Suita, Japan; 8 Wolfson Centre for Prevention of Stroke and Dementia, Nuffield Department of Clinical Neurosciences, University of Oxford, Oxford, United Kingdom; 9 Assistance Publique – Hôpitaux de Paris, Lariboisière Hospital, Translational Neurovascular Centre, Paris, France; 10 Federation Hospitalo–Universitaire NeuroVasc, Université de Paris, Paris, France; 11 INSERM U1141, Paris, France; 12 Department of Neurology, Universitätsmedizin Mannheim, University of Heidelberg, Mannheim, Germany; 13 Division of Neurology, Department of Medicine, The University of Hong Kong, Hong Kong Special Administrative Region, China; 14 Department of Diagnostic Radiology, The University of Hong Kong, Hong Kong Special Administrative Region, China; 15 Department of Neurology, Hospital de la Santa Creu i Sant Pau, Biomedical Research Institute, Barcelona, Spain; 16 Department of Neurosurgery, Kushiro City General Hospital, Kushiro, Japan; 17 A. A. Martinos Center for Biomedical Imaging, Department of Neurology, Department of Radiology, Massachusetts General Hospital, Harvard Medical School, Boston, Massachusetts; 18 Department of Neurology, Heidelberg University Hospital, Heidelberg, Germany; 19 Therese Pei Fong Chow Research Centre for Prevention of Dementia, Gerald Choa Neuroscience Centre, Lui Che Woo Institute of Innovative Medicine, Department of Medicine and Therapeutics, The Chinese University of Hong Kong, Hong Kong Special Administrative Region, China; 20 Department of Neurology, Yonsei University College of Medicine, Seoul, South Korea; 21 Department of Neurology, Seoul Hospital, Ewha Womans University College of Medicine, Seoul, South Korea; 22 Experimental Neurology, Department of Neurosciences, Katholieke Universiteit Leuven, Leuven, Belgium; 23 Department of Neurology, University Hospitals Leuven, Leuven, Belgium; 24 Department of Radiology, Saglık Bilimleri University, Sisli Etfal Education and Research Hospital, Istanbul, Turkey; 25 Department of Neurology, İzmir Katip Çelebi University Atatürk Education and Research Hospital, İzmir, Turkey; 26 Brain Division, Shaare-Zedek Medical Center, Jerusalem, Israel; 27 Faculty of Medicine, Tel Aviv University, Tel Aviv, Israel; 28 Department of Stroke & Neurology, Tel-Aviv Sourasky Medical Center, Tel-Aviv, Israel; 29 Department of Radiology, Saga University Faculty of Medicine, Saga, Japan; 30 Division of Neurology, Department of Internal Medicine, Saga University Faculty of Medicine, Saga, Japan; 31 Calgary Stroke Program, Department of Clinical Neurosciences, Radiology and Community Health Sciences, Hotchkiss Brain Institute, University of Calgary, Calgary, Canada; 32 Department of Neurology and Stroke Centre, University Hospital Basel and University of Basel, Basel, Switzerland; 33 Department of Neurology, University Hospital Inselspital Bern, University of Bern, Bern, Switzerland; 34 Department of Neurology and Neurosurgery, Utrecht University, Utrecht, the Netherlands; 35 Centre for Clinical Brain Sciences, School of Clinical Sciences, University of Edinburgh, Edinburgh, United Kingdom; 36 Lysholm Department of Neuroradiology and the Neuroradiological Academic Unit, Department of Brain Repair and Rehabilitation, University College London Institute of Neurology and the National Hospital for Neurology and Neurosurgery, London, United Kingdom; 37 Liverpool Centre for Cardiovascular Science at University of Liverpool, Liverpool John Moores University and Liverpool Heart and Chest Hospital, Liverpool, United Kingdom; 38 Department of Clinical Medicine, Aalborg University, Aalborg, Denmark; 39 Department of Diagnostic and Interventional Neuroradiology, University Hospital Inselspital Bern, University of Bern, Bern, Switzerland; 40 GHU-Paris Psychiatrie et Neurosciences, Service de Neurologie et Unité Neurovasculaire, Hôpital Sainte Anne, Paris, France; 41 Université de Paris Cité, INSERM U1266, Institute of Psychiatry and Neuroscience of Paris, FHU Neurovasc, Paris, France; 42 GHU-Paris Psychiatrie et Neurosciences, Service de Neuroradiologie, Hôpital Sainte Anne, Paris, France; 43 Université Paris Cité, Institute of Psychiatry and Neuroscience of Paris, INSERM U1266, Paris, France; 44 Peninsula Clinical School, Peninsula Health, Monash University, Melbourne, Australia; 45 Stroke and Ageing Research Group, School of Clinical Sciences at Monash Health, Monash University, Melbourne, Australia; 46 Department of Neurology, University Hospital of Würzburg, Würzburg, Germany; 47 Department of Imaging and Interventional Radiology, Prince of Wales Hospital, The Chinese University of Hong Kong, Hong Kong Special Administrative Region, China; 48 Division of Neurology, Department of Medicine and Therapeutics, Prince of Wales Hospital, The Chinese University of Hong Kong, Hong Kong Special Administrative Region, China; 49 Centre for Clinical Brain Sciences, Edinburgh Imaging, University of Edinburgh, Edinburgh, United Kingdom; 50 UK Dementia Research Institute, University of Edinburgh, Edinburgh, United Kingdom; 51 Institute of Applied Health Sciences, University of Aberdeen, Aberdeen, United Kingdom; 52 Department of Medical Gerontology, Trinity College Dublin, Dublin, Ireland; 53 The Neurovascular Research Unit and Health Research Board, Stroke Clinical Trials Network Ireland, University College Dublin, Dublin, Ireland; 54 Department of Geriatric and Stroke Medicine, Royal College of Surgeons in Ireland University of Medicine and Health Sciences Dublin, Dublin, Ireland; 55 Department of Geriatric and Stroke Medicine, Beaumont Hospital Dublin, Dublin, Ireland; 56 Department of Clinical Neurophysiology, Maastricht University Medical Centre, Maastricht, the Netherlands; 57 Department of Radiology and Nuclear Medicine, Maastricht University Medical Center, Maastricht, the Netherlands; 58 Cardiovascular Research Institute Maastricht, Maastricht University, Maastricht, the Netherlands; 59 Comprehensive Stroke Service, University College London Hospitals NHS Trust, London, United Kingdom; 60 Department of Neurology, Donders Institute for Brain, Cognition and Behaviour, Donders Centre for Medical Neuroscience, Radboud University Medical Center, Nijmegen, the Netherlands; 61 Department of Neurology and Neurorehabilitation, Neurocenter, Lucerne State Hospital, Lucerne, Switzerland; 62 Department of Neurology, National Neuroscience Institute, Singapore, Singapore; 63 Degenerative and vascular cognitive disorders, University of Lille, INSERM, Centre Hospitalier Universitaire de Lille, Lille, France; 64 Department of Radiology, Hospital Foundation Adolphe De Rothschild, Paris, France; 65 Department of Psychiatry and Neuropsychology, Mental Health and Neuroscience Research Institute, Maastricht University, Maastricht, the Netherlands; 66 Department of Neurology, Cardiovascular Research Institute Maastricht School for Cardiovascular Diseases, Maastricht University Medical Centre, Maastricht, the Netherlands; 67 Department of Neurology, The Second Affiliated Hospital of Zhejiang University, School of Medicine, Hangzhou, China; 68 Memory, Aging and Cognition Centre, Yong Loo Lin School of Medicine and Saw Swee Hock School of Public Health, National University of Singapore, Singapore, Singapore; 69 Memory, Aging and Cognition Centre, Yong Loo Lin School of Medicine, National University of Singapore, Singapore, Singapore; 70 Department of Neurology, Research Institute for Medical Innovation, Radboud University Medical Center, Nijmegen, the Netherlands; 71 Department of Epidemiology and Department of Radiology and Nuclear Medicine, ErasmusMC, Rotterdam, the Netherlands; 72 Department of Neurology, Onze Lieve Vrouwe Gasthuis, Amsterdam, the Netherlands; 73 Neurology and Neurorehabilitation, Department of Geriatric Medicine Felix Platter, University of Basel, Basel, Switzerland; 74 Department of Neurology, Kansai Medical University, Osaka, Japan; 75 Memorial Sisli Hospital, Department of Neurology, Istanbul, Turkey; 76 Stroke Division, Florey Institute of Neuroscience and Mental Health, University of Melbourne, Heidelberg, Australia; 77 Department of Neurology, Austin Health, Heidelberg, Australia; 78 Department of Brain Sciences, Imperial College London, London, United Kingdom; 79 Takeda, Cambridge, Massachusetts; 80 New Zealand Brain Research Institute, Christchurch, New Zealand

## Abstract

**Importance:**

Cerebral small vessel disease (SVD) is associated with various cerebrovascular outcomes, but data on sex differences in SVD are scarce.

**Objective:**

To investigate whether the frequency, severity, and distribution of cerebral microbleeds (CMB), other SVD markers on magnetic resonance imaging (MRI), and outcomes differ by sex.

**Design, Setting, and Participants:**

This cohort study used pooled individual patient data from the Microbleeds International Collaborative Network, including patients from 38 prospective cohort studies in 18 countries between 2000 and 2018, with clinical follow-up of at least 3 months (up to 5 years). Participants included patients with acute ischemic stroke or transient ischemic attack with available brain MRI. Data were analyzed from April to December 2023.

**Main Outcomes and Measures:**

Outcomes of interest were presence of CMB, lacunes, and severe white matter hyperintensities determined on MRI. Additionally, mortality, recurrent ischemic stroke, and intracranial hemorrhage during follow-up were assessed. Multivariable random-effects logistic regression models, Cox regression, and competing risk regression models were used to investigate sex differences in individual SVD markers, risk of recurrent cerebrovascular events, and death.

**Results:**

A total of 20 314 patients (mean [SD] age, 70.1 [12.7] years; 11 721 [57.7%] male) were included, of whom 5649 (27.8%) had CMB. CMB were more frequent in male patients, and this was consistent throughout different age groups, locations, and in multivariable models (female vs male adjusted odds ratio [aOR], 0.86; 95% CI, 0.80-0.92; *P* < .001). Female patients had fewer lacunes (aOR, 0.82; 95% CI, 0.74-0.90; *P* < .001) but a higher prevalence of severe white matter hyperintensities (aOR, 1.10; 95% CI, 1.01-1.20; *P* = .04) compared with male patients. A total of 2419 patients (11.9%) died during a median (IQR) follow-up of 1.4 (0.7-2.5) years. CMB presence was associated with a higher risk of mortality in female patients (hazard ratio, 1.15; 95% CI, 1.02-1.31), but not male patients (hazard ratio, 0.95; 95% CI, 0.84-1.07) (*P* for interaction = .01). A total of 1113 patients (5.5%) had recurrent ischemic stroke, and 189 patients (0.9%) had recurrent intracranial hemorrhage, with no sex differences.

**Conclusions and Relevance:**

This cohort study using pooled individual patient data found varying frequencies of individual SVD markers between female and male patients, indicating potential pathophysiological differences in manifestation and severity of SVD. Further research addressing differences in pathomechanisms and outcomes of SVD between female and male patients is required.

## Key Points

**Question:**

Are there differences in the frequency and severity of magnetic resonance imaging markers of cerebral small vessel disease or outcomes among female and male patients with acute ischemic stroke?

**Findings:**

This cohort study using pooled individual patient–data analysis of 38 prospective studies with 20 314 patients with stroke across the world found that female patients had a lower prevalence of cerebral microbleeds and lacunes but higher prevalence of severe white matter hyperintensities.

**Meaning:**

These findings suggest that there are pathophysiological differences in manifestation and severity of cerebral small vessel disease between female and male patients.

## Teaser

This cohort study investigates whether the frequency, severity, and distribution of cerebral microbleeds and other magnetic resonance imaging findings differ by sex in a large combined cohort from 38 studies in 18 countries.

## Introduction

Cerebral small vessel disease (SVD) is a group of disorders affecting small penetrating brain vessels that causes approximately 20% of all ischemic strokes and most intracerebral hemorrhage events and is the most important vascular contributor to cognitive impairment and dementia.^1,2^ Although differences between female and male patients in various aspects of ischemic stroke have been extensively investigated in previous cohort studies,^3,4^ potential sex differences—especially regarding cerebral microbleeds (CMB)—have been less specifically investigated in SVD thus far. While a 2021 meta-analysis showed higher rates of severe SVD in male patients, included studies had various definitions of severity of SVD, and Jiménez-Sánchez et al^5^ identified a lack of sex-stratified data regarding demographics (age), risk factors, and outcomes. Most previous observational studies found no differences regarding the presence of CMB between male and female patients but showed diverging results regarding lacunes, while white matter hyperintensities (WMH) were found to be more severe in female patients.^6,7,8,9^ In cerebral amyloid angiopathy, male patients may have an earlier disease onset and more CMB than female patients.^10^

These sex differences in different SVD markers have generated considerable interest, as this may indicate differences in susceptibility to specific pathophysiological processes, likely not caused by varying exposure to extrinsic risk factors alone. However, most previous studies on sex differences in SVD, and especially CMB, had relevant limitations regarding study size, generalizability, and adjustment for age and comorbidities.^5,6,7,8,9,10^ Therefore, using data from a large international individual patient data analysis with a broad geographic spread, we aimed to investigate sex differences in presence and distribution of CMB and other SVD markers, as well as outcome differences, including recurrent vascular events and mortality.

## Methods

This cohort study was approved by the UK Health Research Authority. Included cohorts obtained ethical and regulatory approvals according to local requirements. Only fully anonymized data were shared, so that individual consent was not required for this individual patient data pooled analysis. This study is reported following the Strengthening the Reporting of Observational Studies in Epidemiology (STROBE) reporting guideline.

We performed a retrospective analysis of pooled individual patient data from the Microbleeds International Collaborative Network (MICON) of prospective observational studies, reported in detail elsewhere.^11^ In short, MICON includes 20 322 patients from 38 cohorts across the world who had an ischemic stroke or transient ischemic attack (TIA) as the index event between 2000 and 2018; magnetic resonance imaging (MRI), including blood-sensitive sequences; and at least 3 months of clinical follow-up. Available patient data included demographics, cerebrovascular risk factors, index event (ischemic stroke or TIA) and Trial of ORG 10172 in Acute Stroke Treatment (TOAST) subtype, MRI findings of SVD, and outcome events. More information on the different cohorts included in the MICON collaboration has been reported elsewhere in detail.^11^ Markers of SVD were assessed on MRI based on the Standards for Reporting Vascular Changes on Neuroimaging (STRIVE) consensus criteria^12^ and included CMB, lacunes, and WMH. WMH were further dichotomized into moderate to severe according to simplified Fazekas scale scores of 2-3 (or equivalents if other scales were used indicating early confluent or confluent WMH) vs none to mild (ie, Fazekas scores 0-1 or equivalents indicating absent or only punctate WMH).^13^ Outcome events assessed were recurrent ischemic stroke (not including TIAs), symptomatic intracranial hemorrhage, and mortality (all-cause mortality) up to 5 years after the index event.

### Statistical Analysis

In this subanalysis of the MICON project, we investigated sex-related differences regarding SVD with a focus on CMB. As first statistical steps, we reported descriptive statistical findings, univariable associations, and associations of covariables corrected for age (an important factor for most risk factors and outcomes and likely to be different between sexes) using random-effects logistic regression models to account for possible differences between study centers.^14^ Then, in multivariable random-effects logistic regression models, we adjusted for age, comorbidities (including vascular risk factors, history of ischemic stroke or intracranial hemorrhage [prior to the index event]), study center location, medication at baseline, index event (ischemic stroke or TIA), stroke etiology according to TOAST criteria, and type of blood-sensitive MRI sequence performed. These factors were determined pre hoc based on previously reported associations and available data in the cohort study. Additionally, we corrected for study site (East Asia vs rest of the world) due to potential differences based on race and ethnicity, as previously performed in this dataset.^15^ We used multiple imputation with chained equations (50 imputations) to account for missing data. Outcome variables (including presence of SVD markers) were not imputed. As a sensitivity analysis, we performed propensity score matching using inverse probability weighting to compare the prevalence of CMB in male and female patients. We further performed subanalyses on the amount of CMB (stratified as 0, 1, 2-4, 5-10, 11-19, and ≥20) and on differences in the presence of lacunes and WMH in patients with CMB. Recurrent ischemic stroke, intracranial hemorrhage, or death during the follow-up period of up to 5 years were investigated using Kaplan-Meier curves and Cox regression (with frailty to account for center effects), again correcting for potential confounders, as previously described. We specifically investigated the associations of sex and the presence of CMB with these outcome events using the aforementioned models and interaction analysis. Regarding the risk of recurrent ischemic stroke and intracranial hemorrhage, we performed a sensitivity analysis using competing risk regression models with death considered as a competing risk and adjusted for clustering.^16^ In addition, we compared unadjusted prevalence of CMB between male and female patients in different age groups using logistic regression to show potential sex differences in CMB presence in age strata.

*P *values were 2-sided, and statistical significance was set at *P* ≤ .05. Analyses were conducted using Stata software version 18 (StataCorp). Data were analyzed from April to December 2023.

## Results

**Figure 1.**
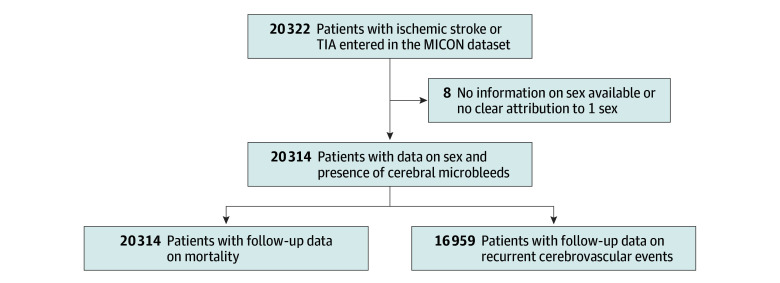
Study Flowchart of Patient Selection MICON indicates Microbleeds International Collaborative Network; TIA, transient ischemic attack.

A total of 20 314 patients (mean [SD] age, 70.1 [12.7] years; 11 721 [57.7%] male) were included in this study (Figure 1). The most prevalent vascular risk factor was hypertension (14 365 patients [70.9%]), followed by hyperlipidemia (7319 patients [41.8%]), atrial fibrillation (8006 patients [39.6%]), and diabetes (4501 patients [24.8%]). Most patients (16 868 patients [83.0%]) had an ischemic stroke as the index event, and 8329 patients (41.0%) were included in an East Asian study center. More detailed baseline information is provided in Table 1.

**Table 1.** Clinical Characteristics of Study Participants Categorized by Sex

Characteristic	Patients, No. (%) (N = 20 314)		*P* value	Age-adjusted *P* value	Missing data, No. (%)
	Female (n = 8593)	Male (n = 11 721)			
Age, mean (SD), y	72.4 (12.9)	68.4 (12.1)	<.001	NA	20 (0.1)
Comorbidities					
Arterial hypertension	6238 (72.8)	8127 (69.5)	<.001	.84	43 (0.2)
Diabetes	1787 (23.3)	2714 (25.9)	<.001	<.001	2146 (10.6)
Hyperlipidemia	3085 (41.5)	4234 (41.9)	.76	.99	2785 (13.7)
Atrial fibrillation	3806 (44.5)	4200 (36.1)	<.001	<.001	105 (0.5)
Ischemic heart disease	909 (11.3)	1699 (15.7)	<.001	<.001	1472 (7.2)
Previous ischemic stroke	1213 (14.3)	1717 (14.8)	.08	<.001	230 (1.1)
Previous intracranial hemorrhage	103 (1.2)	141 (1.2)	.80	.57	543 (2.7)
Current smoker	529 (8.6)	2081 (25.2)	<.001	<.001	5874 (28.9)
East Asian study center	3488 (40.6)	4841 (41.3)	.87	.79	0
Index event					
Ischemic stroke	7076 (82.4)	9792 (83.6)	.002	<.001	11 (0.1)
Transient ischemic attack	1512 (17.6)	1923 (16.4)			
TOAST category					
Large-artery atherosclerosis	957 (20.4)	1795 (26.2)	.001	<.001	8770 (43.2)
Cardioembolism	2034 (43.3)	2525 (36.9)			
Small vessel occlusion	688 (14.6)	1119 (16.4)			
Other determined cause	313 (6.7)	479 (7.0)			
Undetermined cause	710 (15.1)	924 (13.5)			
MRI findings					
Any CMB present	2314 (26.9)	3335 (28.5)	.05	<.001	0
Any lobar CMB	1209 (17.3)	1718 (18.5)	.04	<.001	4004 (19.7)
Any deep CMB	1289 (18.4)	1763 (18.9)	.81	.03	4000 (19.7)
Moderate-to-severe WMH	2105 (38.2)	2344 (32.4)	<.001	.13	7566 (37.2)
Any lacunes	1211 (29.5)	1930 (33.9)	<.001	<.001	10 505 (51.7)
Any cortical superficial siderosis	78 (1.9)	131 (2.3)	.18	.09	10 756 (52.9)
Medication after hospital discharge					
Oral anticoagulation	3535 (41.4)	4202 (35.9)	.03	.99	3 (0.1)
Antiplatelets	5281 (61.5)	8074 (68.9)	<.001	<.001	9 (0.1)
Outcome event					
Intracranial hemorrhage	84 (1.2)	105 (1.1)	.81	.83	3355 (16.5)
Ischemic stroke	467 (6.4)	646 (6.7)	.80	.35	3355 (16.5)
Death	1122 (13.1)	1297 (11.1)	.002	.05	0

Abbreviations: CMB, cerebral microbleed; DOAC, direct oral anticoagulant; MRI, magnetic resonance imaging; NA, not applicable; TOAST, Trial of ORG 10172 in Acute Stroke Treatment; WMH, white matter hyperintensities.

The median (IQR) time from stroke onset to MRI was 2 (0-6) days. There were 5649 patients (27.8%) with at least 1 CMB (deep: 3052 patients [18.7%]; lobar: 2927 patients [17.9%]). Moderate-to-severe WMH were found in 4449 patients (34.9%), lacunes in 3141 patients (32.0%) and cortical superficial siderosis in 209 patients (2.2%). After correction for age, female patients had lower prevalence of diabetes, ischemic heart disease, smoking, and ischemic stroke as index event and higher prevalence of atrial fibrillation and previous ischemic stroke, with no difference regarding the frequency of arterial hypertension (Table 1).

### Cerebral Microbleeds

In univariable random-effects logistic regression models, female patients had a lower prevalence of CMB (2314 female patients [26.9%] vs 3335 male patients [28.5%]; odds ratio [OR], 0.94; 95% CI, 0.88-1.00; *P* = .05). After adjustment for age, this difference was significant (adjusted OR [aOR], 0.85; 95% CI, 0.80-0.91; *P* < .001) (Table 1). There was no difference in the availability of susceptibility-weighted imaging compared with T2*-weighted MRI sequences to determine CMB between male and female patients (female vs male OR, 1.07; 95% CI, 0.96-1.20; *P* = .23).

In a multivariable random-effects logistic regression model adjusting for various baseline variables (age, geographic location; risk factors, including medication at baseline; type and etiology of index event, type of MRI sequence used), female sex was associated with a lower prevalence of CMB (female vs male aOR, 0.86; 95% CI, 0.80-0.92; *P* < .001) (Table 2). Factors associated with a higher prevalence of CMB, next to male sex, included older age, East Asian study center, hypertension, ischemic heart disease, absence of hyperlipidemia, previous ischemic stroke or intracranial hemorrhage, ischemic stroke as index event, small-vessel occlusion as presumed stroke etiology, and use of susceptibility-weighted MRI sequences (Table 2). The estimated intraclass correlation coefficient for this model was 0.053 (95% CI, 0.032-0.087). Differences between female and male patients were consistent with a lower prevalence of lobar CMB (female vs male aOR, 0.84; 95% CI, 0.77-0.91) and deep CMB (female vs male aOR, 0.90; 95% CI, 0.83-0.99) in female patients as well as milder severity (amount) of CMB in female patients (eTable 1 in Supplement 1). In a sensitivity analysis, propensity score matching revealed very similar results regarding the rate of CMB in male and female patients, albeit with a larger CI (female vs male aOR, 0.88; 95% CI, 0.79-0.99).

**Table 2.** Multivariable Random-Effects Regression Model for Odds of Presence of Cerebral Microbleeds (N = 20 314)

Variable	OR (95% CI)	*P* value
Female sex (vs male)	0.86 (0.80-0.92)	<.001
Age, per 1-y increase	1.03 (1.02-1.03)	<.001
Arterial hypertension	1.39 (1.28-1.51)	<.001
Diabetes	0.94 (0.87-1.02)	.14
Hyperlipidemia	0.92 (0.85-0.99)	.02
Atrial fibrillation	0.85 (0.76-0.95)	.005
Ischemic heart disease	1.16 (1.05-1.28)	.003
Previous ischemic stroke	1.41 (1.29-1.54)	<.001
Previous intracranial hemorrhage	2.88 (2.21-3.76)	<.001
Current smoker	0.99 (0.89-1.10)	.88
Ischemic stroke as index event	1.30 (1.17-1.45)	<.001
Susceptibility-weighted imaging performed	1.21 (1.03-1.42)	.02
East Asian study center	1.67 (1.22-2.29)	<.001
Stroke etiology (TOAST classification)		
Large-artery atherosclerosis	1 [Reference]	<.001
Cardioembolism	1.00 (0.88-1.13)	
Small-vessel occlusion	1.27 (1.12-1.43)	
Other determined etiology	1.09 (0.92-1.29)	
Undetermined etiology	1.10 (0.96-1.27)	

Abbreviations: OR, odds ratio; TOAST, Trial of ORG 10172 in Acute Stroke Treatment.

**Figure 2.**
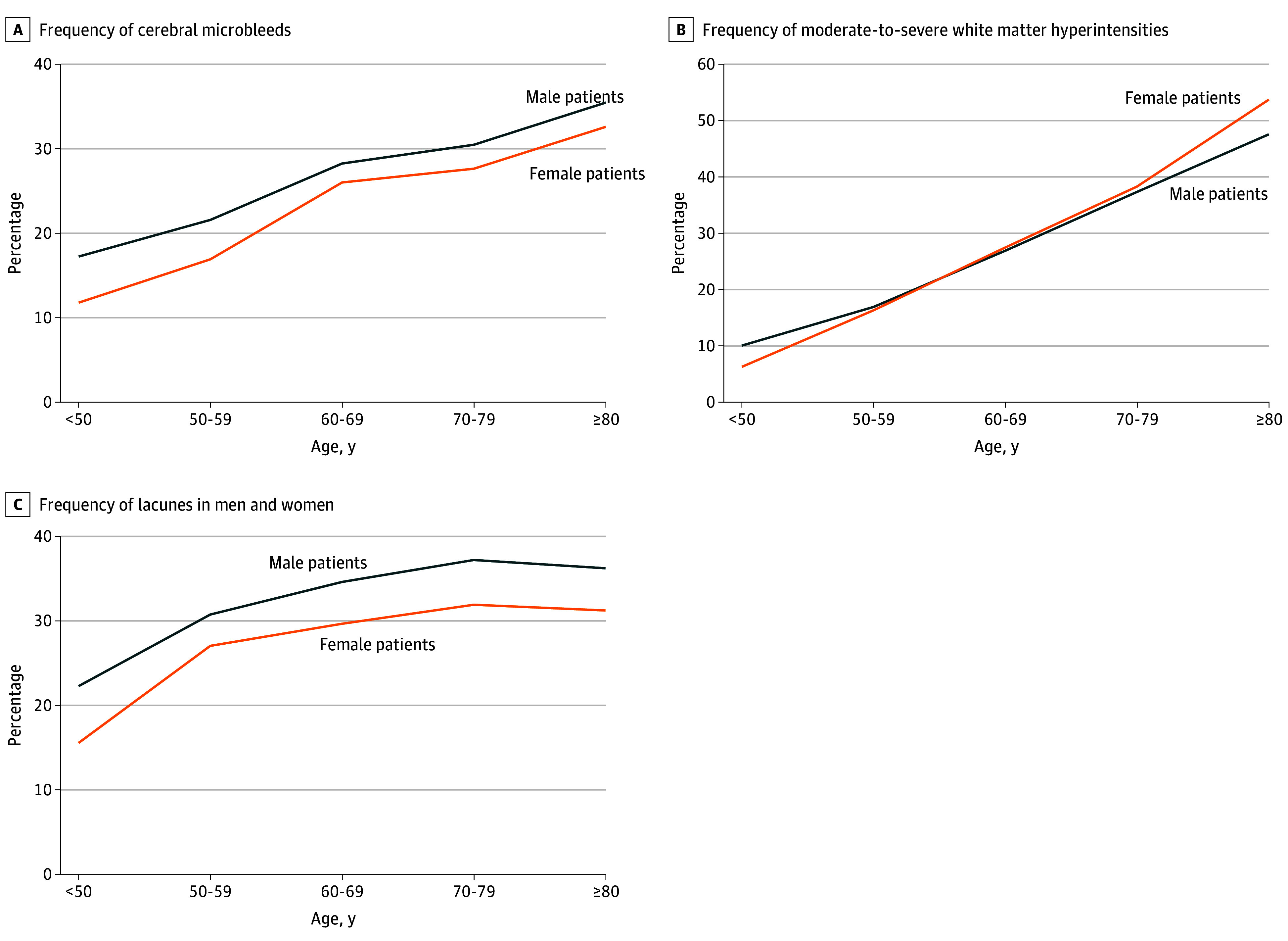
Linear Comparison of Rates of Cerebral Microbleeds, Lacunes, and Moderate-to-Severe White Matter Hyperintensities by Age and Sex

There was no significant interaction between sex and inclusion at an East Asian study center (*P* for interaction = .19). Furthermore, there was no significant interaction regarding the presence of CMB between sex and any other covariable. Although proportions of male and female patients changed across different age groups (with female patients being the minority among younger study participants, but the majority in patients aged ≥80 years), a higher prevalence of CMB in male patients compared with female patients was evident in all age groups, with a larger difference found in younger patients (Figure 2; eTable 2 in Supplement 1).

### Other SVD Markers

Data on lacunes were available in 9809 patients (48.3%) and data on WMH were available in 12 748 patients (62.8%). In multivariable analysis, female patients had a lower prevalence of lacunes (aOR, 0.82; 95% CI, 0.74-0.90; *P* < .001) (eTable 3 in Supplement 1). Cofactors associated with a higher risk for lacunes were older age, hypertension, diabetes, previous ischemic stroke or intracranial hemorrhage, stroke as index event, and small-vessel occlusion as presumed stroke etiology. Female patients had a higher prevalence of moderate-to-severe WMH (female vs male aOR, 1.10; 95% CI, 1.01-1.20; *P* = .04) (eTable 4 in Supplement 1), with older age, hypertension, previous ischemic stroke or intracranial hemorrhage, smoking, and small-vessel occlusion as presumed stroke etiology also associated with higher prevalence of moderate-to-severe WMH.

In a subanalysis restricted to patients with CMB, female patients also had a lower prevalence of lacunes with a similar effect size (female vs male aOR, 0.81; 95% CI, 0.69-0.95; *P* = .01). No difference regarding the prevalence of moderate-to-severe WMH was found (female vs male aOR, 1.04; 95% CI, 0.88-1.23; *P* = .62).

### Recurrent Events and Mortality

**Figure 3.**
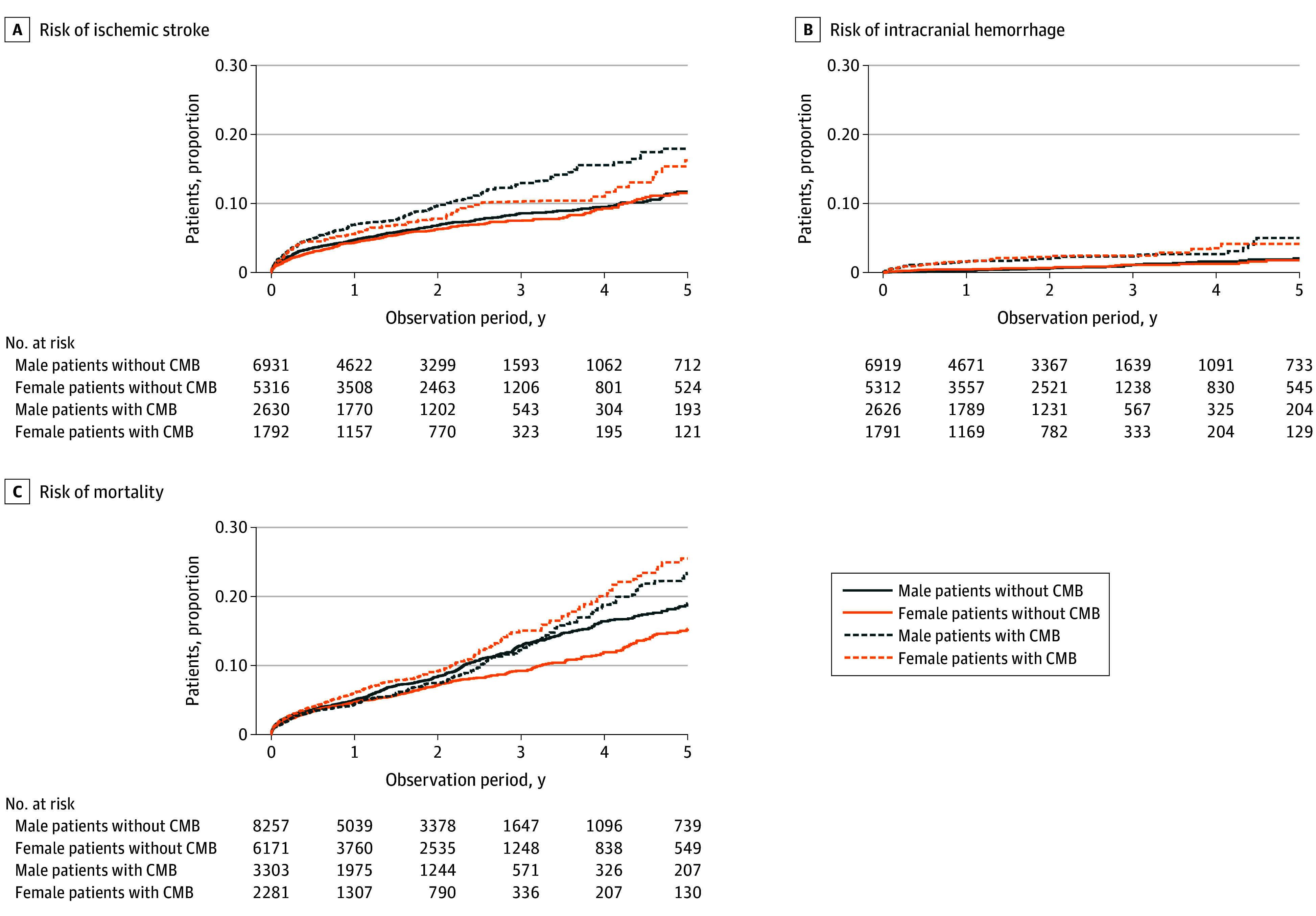
Kaplan-Meier Curves for Recurrent Ischemic Stroke, Intracranial Hemorrhage, and Mortality for Female and Male Patients Without and With Cerebral Microbleeds (CMB), Adjusted for Age

During a median (IQR) follow-up of 2.0 (0.5-2.8) years, 1113 patients (5.5%) had a recurrent ischemic stroke and 189 patients (0.9%) had an intracranial hemorrhage. Data on recurrent ischemic stroke and intracranial hemorrhage were missing in 3355 patients (16.5%). After correction for age, comorbidities, index event type, stroke etiology, anticoagulation or antiplatelet therapy after the index event, and study center setting, there was no difference in ischemic stroke recurrence risk between male and female patients (female vs male hazard ratio [HR], 1.01; 95% CI, 0.89-1.14; *P* = .89). While patients with CMB had a higher risk of recurrent ischemic stroke, this risk was not modified by sex (*P* for interaction = .21) (Figure 3). A sensitivity analysis using a competing-risk regression model yielded very similar results (female vs male HR, 1.01; 95% CI, 0.86-1.20; *P* = .85).

Similarly, there was no difference in the risk of intracranial hemorrhage in the observation period between male and female patients (female vs male HR, 0.96; 95% CI, 0.71-1.30; *P* = .79). Risk for intracranial hemorrhage was higher in patients with CMB, but this was unaffected by sex (*P* for interaction = .50) (Figure 3). Results were essentially unchanged in a competing-risk regression model (female vs male HR, 0.97; 95% CI, 0.71-1.33; *P* = .85).

A total of 2419 patients (11.9%) died over a median (IQR) follow-up of 1.4 (0.7-2.5) years. After correction for relevant factors, female patients had a lower risk of death (female vs male HR, 0.89; 95% CI, 0.82-0.97; *P* = .01). Other characteristics associated with a higher mortality included older age, presence of diabetes, atrial fibrillation, ischemic heart disease, ischemic stroke before the index event, ischemic stroke as index event, and other determined stroke etiology according to the TOAST classification.

The presence of CMB was associated with a higher risk of mortality in female patients (HR, 1.15; 95% CI, 1.02-1.31; *P* = .03) but not male patients (HR, 0.95; 95% CI, 0.84-1.07; *P* = .36). Female patients without CMB had a lower risk of death during the observation period compared with male patients without CMB (HR, 0.83; 95% CI, 0.75-0.92), and although the risk of mortality was increased in female patients with CMB (*P* for interaction = .01), the mortality risk for female patients with CMB did not exceed the risk of male patients with CMB (HR, 1.05; 95% CI, 0.91-1.22) (Figure 3).

## Discussion

In this large cohort study using multinational, pooled, individual patient–data analysis investigating sex differences in SVD markers in patients with ischemic stroke or TIA, we had 3 principal findings. First, CMB were consistently more frequent in male patients and persistent throughout multivariable models, different age groups, and deep or lobar CMB location. Second, other markers of SVD had different prevalence according to sex, with lacunes found more frequently in male patients, and moderate-to-severe WMH more frequently in female patients. Third, CMB was associated with increased risk of mortality in female patients but not male patients.

Most previous neuroimaging studies, including the population-based Rotterdam Scan Study, Northern Manhattan Study, and Atherosclerosis Risk in Communities studies, as well as memory clinic and atrial fibrillation cohorts, found no difference in the frequency of CMB between male and female patients, but these may have been limited due to study size and restricted geographic and/or racial and ethnic spread.^6,7,17,18,19^ The Framingham Heart Study, in which 173 participants (8.9% of the cohort) had CMB, showed a higher prevalence of CMB in male participants, but no multivariable analysis correcting for age and comorbidities was reported.^20^ A 2021 study from the UK Biobank^21^ found CMB in 572 participants (7.0% of the sample), with a higher prevalence of lobar CMB (but not deep or infratentorial CMB) in male participants. In our large sample of 5649 patients with CMB with broad geographic, racial, and ethnic diversity, we might have been able to show potential sex-related differences more clearly than these smaller previous studies. Furthermore, the different study setting (population- or volunteer-based studies as opposed to our study on patients with a recent history of stroke) may have played a relevant role. The higher prevalence of CMB among male patients was persistent throughout age groups (although more marked in patients aged <60 years), study centers, different geographic settings, and after correction for comorbidities (including prior cerebrovascular events), presence of vascular risk factors, stroke etiology, and antithrombotic medication.

The presence of CMB was associated with a higher risk of recurrent cerebrovascular events (both ischemic stroke and intracerebral or intracranial hemorrhage), but not death in the MICON study population.^11^ Interestingly, in this sex-specific analysis, we found that CMB was associated with increased risk of mortality in female patients but not in male patients, a result leaving some room for interpretation. A potential explanation—also based on the lower prevalence of CMB in female patients in general—may be that CMB in female patients indicate more severe SVD, indicating more advanced pathophysiological processes, more severe underlying risk factors, or both, potentially leading to a higher risk of vascular events. However, we did not detect a higher prevalence or severity of other SVD markers in female patients with CMB compared with male patients with CMB. Importantly, we did not find sex differences in recurrent cerebrovascular events according to CMB status.

In our study, male patients also had a higher prevalence of lacunes. Previous studies and meta-analyses have shown a higher prevalence of moderate-to-severe SVD (based on varying definitions) in male patients, especially in those presenting with stroke, as in this study.^5,22^ A Chinese population-based neuroimaging study also showed a higher prevalence of lacunes in male patients (although only age was corrected for).^23^ It should be noted that cerebral SVD is not a singular entity, but a diverse group of disorders, the most frequent being arteriolosclerosis and cerebral amyloid angiopathy. The findings of previous studies and our results suggest that arteriolosclerosis (the main cause of lacunes and deep CMB) is more frequent in male patients than in female patients. It is unclear whether this is due to different exposure to risk factors that we could not correct for, eg, severity and duration of hypertension rather than presence of hypertension alone, body mass index, dietary factors, air pollution, or whether other biological and genetic factors play the main role.

Consistent with previous studies,^8,9^ moderate-to-severe WMH were more frequent in female patients in our analysis. This again points to differences in pathophysiological processes and it appears not simply that all SVD is more severe in male patients, but sex-related differences are much more complex, which has also been indicated in a 2022 large meta-analysis^24^ that identified sex differences in circulating metabolites in association with WMH in a general population of middle-aged and older adults.

The core strength of this study is the large study size and wide geographic spread, allowing for better statistical power, precision, generalizability, and adequate adjustment for age and comorbidities. Moreover, the availability of brain MRI allowed us to sensitively detect and rate the severity of different SVD features, including CMB.

### Limitations

This study has some limitations. A selection bias of individual study cohorts cannot be excluded due to the study design, with a slight preponderance of male patients included in the study. Analysis of SVD markers other than CMB was somewhat limited by missing data. We were also not able to calculate a common total SVD burden score,^22^ as we had no information on enlarged perivascular spaces in most patients. While most important vascular risk factors could be considered in this study, there were no available data on stroke severity, causes of death, or lifestyle-related risk factors, such as diet and physical activity. We were able to consider geographic differences (as we analyzed data from cohorts across 4 continents) but did not have data on patient race and ethnicity available. Additionally, we were not able to investigate potential disparities in long-term medical management and social determinants of health, which might have also played a relevant role regarding analyzed outcome events.

## Conclusions

This large cohort study including international pooled analysis of patients with a recent ischemic cerebrovascular event found a higher prevalence of CMB and lacunes in male patients, but a higher prevalence of moderate-to-severe WMH in female patients. Our study found further sex differences in SVD markers on brain MRI after correction for important risk factors, including an association of CMB with an increased risk of mortality in female patients but not male patients. These findings are relevant for future research, which should address sex-specific differences in the pathophysiology and outcome of cerebrovascular disease and SVD.

## Notes

Supplement 1. **eTable 1.** Multivariable Logistic Regression Model Regarding Severity of Cerebral Microbleeds (Stratified as 0, 1, 2-4, 5-9, 10-19 and ≥20) **eTable 2.** Univariable Comparison of Cerebral Microbleed Presence in Men and Women of Different Age Groups **eTable 3.** Multivariable Random-Effects Regression Model Regarding Presence of Lacunes **eTable 4.** Multivariable Random-Effects Regression Model Regarding Presence of Moderate-to-Severe White Matter Hyperintensities

Supplement 2.
Data Sharing Statement
