# Error in Author Name

**DOI:** 10.1001/jamanetworkopen.2025.1456

**Published:** 2025-02-20

In the Original Investigation titled “Sex Differences in Frequency, Severity, and Distribution of Cerebral Microbleeds,”^1^ published October 15, 2024, Dr Al-Shahi Salman’s name appeared incorrectly in the Article Information. This article has been corrected.^1^
