# Supplementary material for: Sex Differences in Frequency, Severity, and Distribution of Cerebral Microbleeds
Source: JAMA Netw Open. 2024 Oct 15;7(10):e2439571. doi: 10.1001/jamanetworkopen.2024.39571 (PMC11581520; doi:10.1001/jamanetworkopen.2024.39571)
Supplement: Supplement 1. — eTable 1. Multivariable Logistic Regression Model Regarding Severity of Cerebral Microbleeds (Stratified as 0, 1, 2-4, 5-9, 10-19 and ≥20) eTable 2. Univariable Comparison of Cerebral Microbleed Presence in Men and Women of Different Age Groups eTable 3. Multivariable Random-Effects Regression Model Regarding Presence of Lacunes eTable 4. Multivariable Random-Effects Regression Model Regarding Presence of Moderate-to-Severe White Matter Hyperintensities [file jamanetwopen-e2439571-s001.pdf]

Supplemental Online Content

Fandler-Höfler S, Eppinger S, Ambler G, et al; Microbleeds International Collaborative Network. Sex differences in frequency, severity, and distribution of cerebral microbleeds. *JAMA Netw Open*. 2024;7(10):e2439571. doi:10.1001/jamanetworkopen.2024.39571

eTable 1. Multivariable Logistic Regression Model Regarding Severity of Cerebral Microbleeds (Stratified as 0, 1, 2-4, 5-9, 10-19 and ≥20)
eTable 2. Univariable Comparison of Cerebral Microbleed Presence in Men and Women of Different Age Groups
eTable 3. Multivariable Random-Effects Regression Model Regarding Presence of Lacunes
eTable 4. Multivariable Random-Effects Regression Model Regarding Presence of Moderate-to-Severe White Matter Hyperintensities

This supplemental material has been provided by the authors to give readers additional information about their work.

© 2024 Fandler-Höfler S et al. *JAMA Network Open.*

Supplementary table 1: Multivariable logistic regression model regarding severity of cerebral microbleeds (stratified as 0, 1, 2-4, 5-9, 10-19 and ≥20; n=20223)

Variable	Odds Ratio (95% Confidence Interval)	p-value
Female sex (vs. male)	0.86 (0.79-0.93)	<0.001
Age, per 1-year increase	1.02 (1.02-1.03)	<0.001
Arterial hypertension	1.40 (1.21-1.62)	<0.001
Diabetes	0.96 (0.88-1.04)	0.29
Hyperlipidemia	0.89 (0.81-0.97)	0.008
Atrial fibrillation	0.73 (0.59-0.91)	0.006
Ischemic heart disease	1.19 (1.05-1.34)	0.006
Previous ischemic stroke	1.46 (1.33-1.61)	<0.001
Previous intracranial hemorrhage	3.22 (2.20-4.71)	<0.001
Current smoker	0.97 (0.87-1.08)	0.56
Ischemic stroke as index event	1.50 (1.22-1.86)	<0.001
Susceptibility-weighted imaging performed	1.18 (0.84-1.67)	0.33
East Asian study center	1.55 (1.16-2.08)	0.003
Stroke etiology (TOAST classification):		
Large-artery atherosclerosis	Ref	
Cardioembolism	0.86 (0.69-1.06)	0.16
Small-vessel occlusion	1.26 (1.06-1.51)	0.01
Other determined etiology	1.19 (1.00-1.43)	0.05
Undetermined etiology	1.01 (0.82-1.25)	0.90

© 2024 Fandler-Höfler S et al. *JAMA Network Open.*

Supplementary table 2: Univariable comparison of cerebral microbleed presence in men and women of different age groups (n=20314)

	**Cerebral microbleeds present**	**Odds Ratio for men (95% CI)**
Men, age <50 years	140/812 (17.2%)	1.56 (1.13-2.15)
Women, age <50 years	61/518 (11.8%)	
Men, age 50-59 years	399/1849 (21.6%)	1.35 (1.09-1.67)
Women, age 50-59 years	141/834 (16.9%)	
Men, age 60-69 years	886/3137 (28.2%)	1.12 (0.98-1.28)
Women, age 60-69 years	435/1673 (26.0%)	
Men, age 70-79 years	1138/3735 (30.5%)	1.15 (1.03-1.28)
Women, age 70-79 years	760/2751 (27.6%)	
Men, age ≥80 years	772/2177 (35.5%)	1.14 (1.01-1.28)
Women, age ≥80 years	915/2808 (32.6%)	

© 2024 Fandler-Höfler S et al. *JAMA Network Open.*

Supplementary table 3: Multivariable random-effects regression model regarding presence of lacunes (n=9809)

**Variable**	**Odds Ratio (95% Confidence Interval)**	**p-value**
Female sex (vs. male)	0.82 (0.74-0.90)	<0.001
Age, per 1-year increase	1.02 (1.02-1.03)	<0.001
Arterial hypertension	1.36 (1.22-1.52)	<0.001
Diabetes	1.16 (1.05-1.29)	0.005
Hyperlipidemia	1.04 (0.94-1.15)	0.48
Atrial fibrillation	0.62 (0.53-0.72)	<0.001
Ischemic heart disease	1.04 (0.90-1.21)	0.60
Previous ischemic stroke	2.16 (1.91-2.44)	<0.001
Previous intracranial hemorrhage	1.67 (1.21-2.30)	0.002
Current smoker	1.07 (0.94-1.22)	0.29
Ischemic stroke as index event	1.43 (1.21-1.70)	<0.001
East Asian study center	1.81 (0.69-4.73)	0.23
Stroke etiology (TOAST classification):		<0.001
Large-artery atherosclerosis	Ref	
Cardioembolism	0.94 (0.79-1.11)	
Small-vessel occlusion	1.33 (1.15-1.54)	
Other determined etiology	0.91 (0.70-1.18)	
Undetermined etiology	0.94 (0.79-1.11)	

© 2024 Fandler-Höfler S et al. *JAMA Network Open.*

Supplementary table 4: Multivariable random-effects regression model regarding presence of moderate-to-severe white matter hyperintensities (n=12748)

Variable	Odds Ratio (95% Confidence Interval)	p-value
Female sex (vs. male)	1.10 (1.01-1.20)	0.04
Age, per 1-year increase	1.08 (1.07-1.08)	<0.001
Arterial hypertension	1.45 (1.31-1.61)	<0.001
Diabetes	1.05 (0.95-1.16)	0.34
Hyperlipidemia	0.95 (0.86-1.04)	0.26
Atrial fibrillation	0.93 (0.81-1.07)	0.31
Ischemic heart disease	1.04 (0.92-1.17)	0.54
Previous ischemic stroke	1.46 (1.31-1.64)	<0.001
Previous intracranial hemorrhage	2.68 (1.88-3.83)	<0.001
Current smoker	1.19 (1.04-1.37)	0.01
Ischemic stroke as index event	1.12 (0.97-1.29)	0.12
East Asian study center	1.86 (0.77-4.50)	0.17
Stroke etiology (TOAST classification):		<0.001
Large-artery atherosclerosis	Ref	
Cardioembolism	0.89 (0.76-1.05)	
Small-vessel occlusion	1.23 (1.04-1.44)	
Other determined etiology	0.92 (0.72-1.18)	
Undetermined etiology	0.97 (0.81-1.17)	

© 2024 Fandler-Höfler S et al. *JAMA Network Open.*
