# Supplementary material for: Sex Differences in Frequency, Severity, and Distribution of Cerebral Microbleeds
Source: JAMA Netw Open. 2024 Oct 15;7(10):e2439571. doi: 10.1001/jamanetworkopen.2024.39571 (PMC11581520; doi:10.1001/jamanetworkopen.2024.39571)
Supplement: Supplement 2. — Data Sharing Statement [file jamanetwopen-e2439571-s002.pdf]

Data Sharing Statement

Fandler-Höfler. Sex Differences in Frequency, Severity, and Distribution of Cerebral Microbleeds. *JAMA Netw Open*. Published October 15, 2024.
doi:10.1001/jamanetworkopen.2024.39571

Data
Data available: Yes
Data types: Deidentified participant data
How to access data: For access to the dataset generated during this study, please contact the corresponding author. Requests will be discussed in the steering group of the MICON collaboration and decided on a case-to-case basis.
When available: With publication

Supporting Documents
Document types: None

Additional Information
Who can access the data: Requests will be discussed in the steering group of the MICON collaboration and decided on a case-to-case basis.
Types of analyses: Requests of data for any purpose will be discussed in the steering group of the MICON collaboration and decided on a case-to-case basis.
Mechanisms of data availability: Data will be made available with investigator support based on decisions of the steering group of the MICON collaboration.
